# Thymoquinone and cisplatin as a therapeutic combination in lung cancer: *In vitro and in vivo*

**DOI:** 10.1186/1756-9966-29-87

**Published:** 2010-07-01

**Authors:** Syed H Jafri, Jonathan Glass, Runhua Shi, Songlin Zhang, Misty Prince, Heather Kleiner-Hancock

**Affiliations:** 1 Feist-Weiller Cancer Center, Louisiana State University, 1501 Kings Highway, Shreveport LA, 71130 USA; 2 Department of Medicine, Louisiana State University, 1501 Kings Highway, Shreveport LA, 71130 USA; 3 Department of Pathology, Louisiana State University, 1501 Kings Highway, Shreveport LA, 71130 USA; 4 Department of Pharmacology, Toxicology and Neuroscience, Louisiana State University, 1501 Kings Highway, Shreveport, LA, USA

## Abstract

**Background:**

Thymoquinone (TQ) is a compound extracted from Black Caraway seeds of *Nigella Sativa *and is active against various cancers. Cisplatin (CDDP) is the most active chemotherapeutic agent in Lung Cancer. Here we report activity of TQ against non-small cell lung cancer (NSCLC) and small cell lung cancer (SCLC) cell lines alone and in combination with Cisplatin (CDDP).

**Methods:**

For proliferation MTT assay, cell viability trypan blue assay and for apoptosis Annexin-V FITC assay were used in NCI-H460 and NCI-H146 cell lines. Inhibition of invasion by TQ was assessed using Matrigel assay and its affect on release of various cytokines was determined using RayBio Human Cytokine detection kit. Mouse xenograft model using NCI-H460 was used to determine *in vivo *activity of TQ and CDDP. Inhibition of LPS induced NF-κB expression by TQ was determined using transgenic mice expressing a luciferase reporter.

**Results:**

TQ was able to inhibit cell proliferation, reduce cell viability and induce apoptosis. TQ at 100 μM and CDDP at 5 μM inhibited cell proliferation by nearly 90% and the combination showed synergism. TQ was able to induced apoptosis in both NCI-H460 and NCI-H146 cell lines. TQ also appears to affect the extracellular environment inhibiting invasion and reducing the production of two cytokines ENA-78 and Gro-alpha which are involved in neo-angiogenesis. Using a mouse xenograft model we were able to demonstrate that combination of TQ and CDDP was well tolerated and significantly reduced tumor volume and tumor weight without additional toxicity to the mice. In the combination arms (TQ5 mg/kg/Cis 2.5 mg/kg) tumor volume was reduced by 59% and (TQ20 mg/kg/Cis 2.5 mg/kg) by 79% as compared to control which is consistent with *in vitro *data. TQ down regulated NF-κB expression which may explain its various cellular activities and this activity may prove useful in overcoming CDDP resistance from over expression of NF-κB.

**Conclusions:**

Thus TQ and CDDP appear to be an active therapeutic combination in lung cancer.

## Background

Lung cancer is the leading cause of cancer related death in United States. In the US alone it is estimated that in the year 2008, approximately 215,020 new cases of lung cancer were diagnosed and an estimated 161,480 deaths were reported. The mortality from lung cancer is more than the combined mortality from breast, prostate and colorectal cancers [1].

The two major histological types of lung cancer are non-small cell lung cancer (NSCLC) accounting for about 85% of cases and small cell lung cancer (SCLC) accounting for 15% of cases [2]. Approximately 16% of NSCLC patients are diagnosed with early stage or localized disease and are treated with surgical resection [3]. Systemic chemotherapy is indicated in adjuvant treatment [4] as well as in advanced stages of NSCLC and is also used in treatment of all stages of SCLC. The most active chemotherapeutic agent for the treatment of NSCLC and SCLC is cisplatin (CDDP) which is used in a doublet with other agents such as paclitaxel, gemcitabine and docetaxel [5]. The response rate in NSCLC from CDDP alone is about 20% and in combination with a second agent improves to about 26% [6].

Recently, new agents have been approved for treatment of lung cancer including erlotinib [7] and bevacizumab [8]. However the overall 5 year survival from lung cancer has not changed appreciably in the past 25 years and remains dismal at 16% [1]

The Black Caraway seed also known as (*Nigella Sativa*, Ranunculaceae family), is an annual herb that grows in countries bordering Mediterranean Sea, Pakistan and India.

The seed has been used as a natural remedy for more than 2000 years to promote health and treat diseases. Medicinal properties of black seeds have even been mentioned by the Prophet of Islam, Muhammad (Peace be upon him) and its use was recommended for various ailments [9].

Thymoquinone (TQ) is the bioactive constituent of the volatile oil of black seed. It has been shown to exert anti-inflammatory, anti-oxidant and anti-neoplastic effects both *in vitro *and *in vivo *[10]. TQ has been shown to exhibit anti-tumor activities on cells lines derived from ovarian, breast and colon cancers [11].

TQ has also been shown to potentiate the anti-tumor activity of CDDP in Ehrlic ascites sarcoma (EAC) and simultaneously protected against CDDP nephrotoxicity [12]. Using both mouse and other rodent models it was shown that TQ when administered orally after mixing in drinking water ameliorated the nephrotoxicity from CDDP and also improved CDDP therapeutic index.

Combining the most active chemotherapeutic drugs with agents that target specific pathways offers a powerful approach to cancer treatment and may counteract the many ways that human cancer cells can become drug resistant. The platinum atom of CDDP forms covalent bonds to the N7 positions of purine bases to afford primarily 1, 2- or 1, 3-intra strand cross links and a lower number of inter strand cross links which eventually leads to apoptosis [13]. There is evidence that CDDP induces increased expression of NF-κB and that this activity results in increased CDDP resistance [14].

NF-κB controls cellular proliferation in part by increasing expression of cyclin D1 which moves cells from G1 to S phase [15]. TQ has been reported to suppress tumor necrosis factor (TNF) induced NF-κB expression in human chronic myeloid leukemia cells (KBM-5) which may also explain why cells undergo apoptosis [16]. TQ was shown to suppress expression of NF-κB activation pathway through modulation of p65 subunit of NF-κB and inhibition of IκBα kinase (IKK) [16].

Thus in the present study we have combined a non-cell cycle specific active chemotherapy drug CDDP which causes direct DNA damage with another agent TQ which targets the cell cycle at the transition from G1 to S phase hypothesizing the combination of TQ and CDPP will enhance the efficacy of CDDP and possibly overcome its resistance by suppression of CDDP induced over expression of NF-κB. TQ by suppressing NF-κB, should also affect tumor angiogenesis and metastasis [15]

## Materials and methods

### In Vitro experiments

#### Cell culture

NSCLC cell line NCI-H460 was generously provided by Dr James A. Cardelli (Louisiana State University Health Sciences Center, Shreveport, LA). SCLC cell line NCI-H146 was purchased from American Type Culture Collection (ATCC). Cells were grown in RPMI 1640 (Cell gro) supplemented with 10% Fetal bovine serum (FBS), 1% Penicillin and Streptomycin in a humidified incubator with 5% CO2 at 37°C.

#### 1) Cell proliferation assay

NCI-H460 cells (NSCLC cell line) were seeded at a density of 5,000 cells per well in 96 well plates and after 24 hrs cells were treated with 80 μM and 100 μM Thymoquinone (TQ) (Sigma Aldrich, St Louis MO) in 0.1% DMSO, 1.25 μM, 2.5 μM and 5.0 μM Cisplatin (CDDP) (Sigma Aldrich, St Louis MO) or TQ and CDDP at various combinations as noted. These doses of TQ and CDDP were chosen based on IC50 calculated from earlier experiments (Results not shown). There were four wells per condition and experiment was repeated twice to validate results. MTT (3-(4,5 Dimethylthiazol -2-yl)-2,5-dipheynyltetrazolium bromide) assay was used to determine live cells at 24, 48 and 72 hrs after treatment as per the manufacturer's (Invitrogen) instructions.

#### 2) Cell viability assay

Cell viability of the SCLC cell line NCI-H146 was assessed using the trypan blue cell viability assay. About 5,000 cells/well were seeded in 6 well plates using appropriate media and left in incubator overnight. At 24 hrs cells were treated with TQ at doses 20, 40, 60, 80 and 100 μM with appropriate DMSO concentration as the control. Cells were collected 2 hours later by low speed centrifugation and trypan blue viability assay was performed with the aid of a Coulter counter.

#### 3) Apoptosis assay

Apoptosis in the NCI-H460 and NCI-H146 cell lines was detected using Annexin-V FITC Apoptosis detection kit I (BD Pharmingen). 24 hrs after treatment with 100 μM TQ both cell lines were removed from the plates using trypsin in the case of NCI-H460 only. Cells were extensively washed with PBS and adjusted to 1 × 10^6 ^cells/ml and stained with Annexin V FITC and propidium iodine as per the manufacture's instruction. Presence of apoptosis was detected using a Cytomics FC 500 Beckman Coulter Flowcytometry (Coulter, Inc, Hialeah Fl).

#### 4) Cytokine Assay

The effect of TQ on release of cytokines was assessed using the RayBio Human Cytokine Antibody Array C Series 2000. (RayBio Tech. Inc. Norcross, GA). Cells grown in serum free media in 12 well plates at a density of 5,000 cells/well were treated with DMSO or TQ 100 μM and the media collected after 24 hours. The collected media was applied on cytokine membranes which were then exposed to a photographic film for approximately 30 minutes after which the films were developed in a dark room. The resulting images were analyzed using Image J Software to measure expression of various cytokines.

#### 5) Invasion assay

The effect of TQ on tumor cell invasion was assayed using a Matrigel based assay. Trans well inserts (Corning Life Science, Corning, NY) with 8 micron diameter pores were coated with 20 μL of Matrigel (BD Biosciences), dried, and subsequently rehydrated first using 750 μL of serum free medium, followed by the addition of complete medium. NCI-H460 cells at a density of 25,000 cells in 100 μL per insert were applied. After 2 hrs cells were treated with DMSO or TQ at 20, 40 or 80 μM. After 24 hrs the non-invasive cells were removed and the cells that had invaded into the Matrigel were detected by fixation with 10% neutral buffered formalin followed by staining with hematoxylin. Membranes were removed from inserts, mounted on slides and invading cells counted using a microscope with a 40× objective.

#### 6) *In Vivo *Experiments

##### a) **Animals**

5-6 weeks old female Severe combined immunodeficiency mice (SCID) mice were obtained from Harlan Laboratory (Harlan Laboratories, Inc, Indianapolis, IN) and maintained under pathogen free conditions in a temperature and humidity controlled animal care facility with a 12 hours light dark cycle. The animal protocol for this experiment has been approved by the Committee of Animal Care and Use, LSUHSC in accordance with National Institutes of Health (NIH) guidelines. All procedures were conducted under sterile conditions. Mice were allowed access to sterile food and water *ad libitum*.

##### b) Maximum Tolerated Dose

Groups of 3 or 4 female SCID mice were randomized to receive TQ alone or in combination with CDDP. TQ was prepared by dissolving in cremophor: ethanol: PBS (1:1:4) and CDDP was prepared by dissolving in PBS. TQ was given at 5, 10 and 20 mg/kg subcutaneously (s.c.) on Monday, Wednesday and Friday for 3 weeks either alone or in combination with CDDP at 5 mg/kg i.p once a week for 3 weeks. MTD was considered the highest dose in which no mortality was observed. At the end of three weeks mice were sacrificed and liver and kidneys were harvested for histological analysis.

##### c) Mouse xenograft experiment

For the xenograft study, female SCID mice (age 5-6 weeks old) were shaved on the flank two days prior to injection with tumor cells. Xenografts were obtained by injecting NCI-H460 2 × 10^6 ^cells subcutaneously into the right flank of each mouse. Tumors were allowed to grow for one week and when tumor volume reached approximately 20 mm^3 ^mice were randomized to 6 groups with 10 mice in each group. Tumor volume was calculated using the formula V = (L × W^2^) × 0.5 where V= volume, L = length, W = width.

Mice were randomized into following 6 treatment groups with 10 mice in each group and treated for 3 weeks.

1) Control

2) TQ alone 5 mg/kg (s.c) M, W, F

3) TQ alone 20 mg/kg (s.c) M, W, F

4) CDDP alone 2.5 mg/kg (i.p) every Monday

5) CDDP 2.5 mg/kg+TQ5 mg/kg (combination)

6) CDDP 2.5 mg/kg+TQ20 mg/kg (combination)

Tumor volume and body weight was measured M, W, F for three weeks during the course of study. At the end of three weeks (Day 26) mice were sacrificed by CO2 asphyxiation in a pre charged chamber and tissue samples were obtained for histological analysis. Mean tumor weight was also calculated after harvesting tumors.

#### 7) NF-κB expression in lung cancer xenografts

NF-κB in the xenografts was assayed by Western blot analysis on snap-frozen xenograft tumor specimens which were pooled in duplicate for a total of 5 samples per group. Briefly, aliquots of the xenograft samples were lysed in Radio-Immunoprecipitation Assay_RIPA (Buffer) containing protease and phosphatase inhibitor cocktails and EDTA (Thermo Scientific, Rockford, IL). Proteins (40 μg) were resolved by SDS-PAGE [17] and the proteins were transferred electrophoretically to PVDF membranes (0.45 μm, Millipore Corp., Billerica, MA) [18]. Western blot assays were conducted using antibodies against phospho-Ser529 NF-κB (1:200) and unphosphorylated NF-κB (1:500), followed by the appropriate secondary antibodies and enhanced chemiluminescent detection [19]. The intensities of immunostained proteins were determined using Image J software http://rsb.info.nih.gov/ij/ mean intensities calculated as a ratio of phospho-NF-κB to total NF-κB, and normalized to Hela cell lysates run on the same gel.

#### 8) NF-κB suppression by TQ

We assessed suppression of NF-κB by TQ using the light producing animal model (LPTA) NF-κB -RE-luc (Oslo) which is a transgenic mice expressing a luciferase reporter whose transcription is dependent on NF-κB [20]. The luminescence from luciferase can be detected real time using an ultrasensitive camera IVIS 100 Imaging system (Caliper Life sciences, Hopkinton MA). Lipopolysaccharide (LPS) or Tumor necrosis factor-alpha (TNF-α) are used to induce NF-κB activity. Initially 5-8 mice/group were injected with either vehicle alone or TQ 5 mg/kg or 20 mg/kg subcutaneously and images obtained to detect any effect of TQ on NF-κB expression with 2.5 mg D-luciferin substrate administered 15 minutes prior to each imaging without prior induction with LPS. Two days later mice were injected with vehicle or 5 mg/kg or 20 mg/kg TQ subcutaneously, followed 30 minutes later by injection of LPS (2.7 mg/kg i.p) with mice then imaged at 3 hrs and 24 hrs interval to assess NF-κB activity with 2.5 mg D-luciferin substrate administered 15 minutes prior to each imaging. The luminescence intensity was quantitated in regions of interest (ROI) using Living Image^® ^3.0 software (Caliper Life Sciences, Inc. Hopkinton, MA).

### Statistical analysis

For the MTT assay factorial analyses of variance (ANOVA) were used to determine the effect of TQ, CDDP and control with the time. Student-Newman-Keuls test was used to determine statistical significance with P value < 0.05 considered significant.

For the mouse xenograft studies and for NF-κB expression using the luciferase reporter mouse SAS^® ^Proc Mixed was used and least squares means (LS-means) were estimated. The Bonferroni method was used for multiple comparisons adjustments on the differences of LS-means.

## Results

### 1) TQ inhibits proliferation alone and in combination with CDDP

In the MTT assay TQ at 80 and 100 μM showed significant inhibition of cell proliferation most noticeable at 24 hrs. The effect of TQ alone on cell proliferation waned with time with less activity observed at 48 and 72 hrs suggesting more frequent dosing of TQ may be required to demonstrate a sustained effect. CDDP alone at 24 hrs was not every active as compared to TQ but at 48 and 72 hrs showed significant inhibition of cell proliferation.

**Figure 1:**
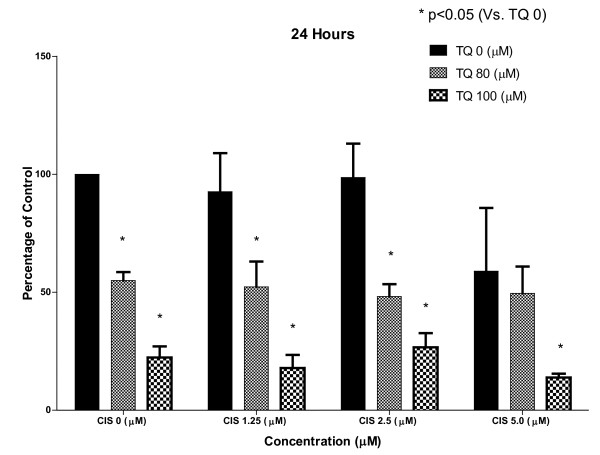
**The figure shows results of MTT assay for cell proliferation using NSCLC cell line NCI-H460 at 24, 48 and 72 hrs with control group representing 100% cell proliferation depicted by extreme left solid line**. TQ alone is more active at 24 hrs and CDDP more active at 48 and 72 hrs. The combination of TQ and CDDP is more active than each agent alone with up to 89% inhibition of cell proliferation at 72 hrs with combination of TQ 100 μM and CDDP 5 μM. (*) shows significant inhibition by TQ at each level of CDDP (p < 0.05).

**Figure 2:**
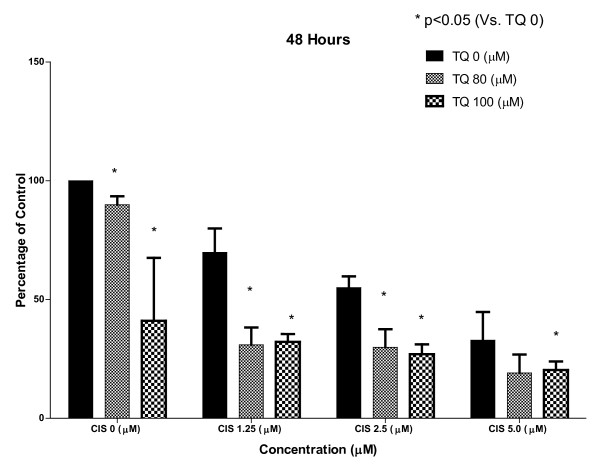
**The figure shows results of MTT assay for cell proliferation using NSCLC cell line NCI-H460 at 24, 48 and 72 hrs with control group representing 100% cell proliferation depicted by extreme left solid line**. TQ alone is more active at 24 hrs and CDDP more active at 48 and 72 hrs. The combination of TQ and CDDP is more active than each agent alone with up to 89% inhibition of cell proliferation at 72 hrs with combination of TQ 100 μM and CDDP 5 μM. (*) shows significant inhibition by TQ at each level of CDDP (p < 0.05).

**Figure 3:**
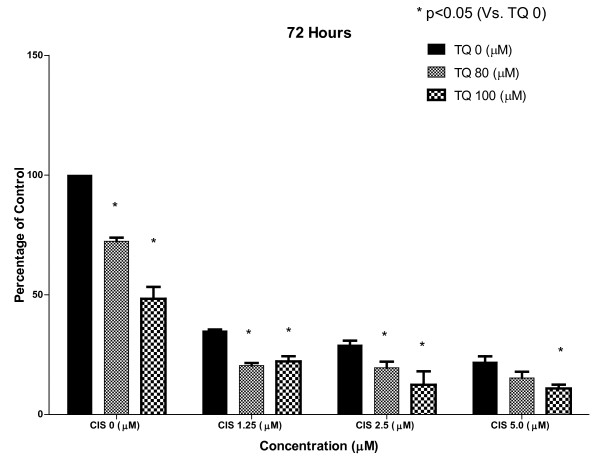
**The figure shows results of MTT assay for cell proliferation using NSCLC cell line NCI-H460 at 24, 48 and 72 hrs with control group representing 100% cell proliferation depicted by extreme left solid line**. TQ alone is more active at 24 hrs and CDDP more active at 48 and 72 hrs. The combination of TQ and CDDP is more active than each agent alone with up to 89% inhibition of cell proliferation at 72 hrs with combination of TQ 100 μM and CDDP 5 μM. (*) shows significant inhibition by TQ at each level of CDDP (p < 0.05).

The combined effect of TQ and CDDP on cell proliferation was most noticeable at 48 and 72 hrs with 89% inhibition of cell proliferation observed at 72 hrs (Figure 1, Figure 2, Figure 3)

### 2) TQ enhances the effect of Cisplatin with synergism between the two agents

**Figure 4:**
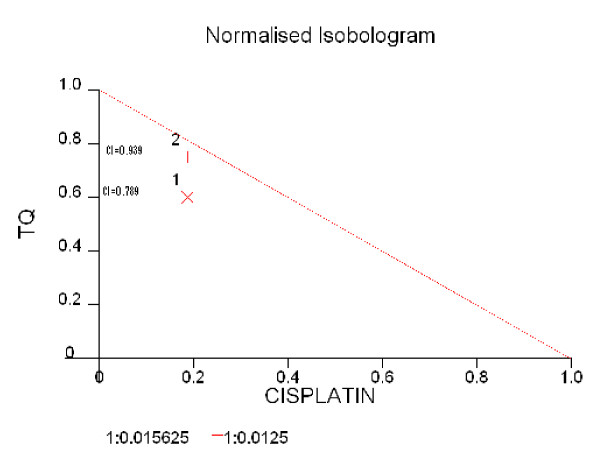
**Combination Index (CI) between TQ and CDDP at 72 hrs using NCI-H460**. CI 0.789 at TQ 80 μMolar and CDDP 1.25 μMolar. CI 0.939 at TQ 100 μMolar and CDDP 1.25 μMolar. The Combination Index (CI) was calculated using *Calcusyn *software with CI of <1 suggesting synergism between TQ and CDDP using cell line NCI-H460.

When the NCI-H460 cells were grown in the presence or absence of TQ and CDDP it was apparent that the combined effect of TQ and CDDP was more than the each agent alone. To confirm the presence of synergism we determined the Combination index (CI) for two combination treatment groups using *Calcuysyn software *with CI < 1 indicating synergism, CI > 1 indicating antagonism and CI = 1 indicating an additive effect. Synergism was most noticeable at 72 hrs in the groups TQ 80 and CDDP 1.25 (CI = 0.789) (Figure 4) as well as TQ 100 and CDDP 2.5 (CI = 0.761).

The combination of TQ (100 μM) and CDDP (5 μM) at 72 hrs showed 89% inhibition of cell proliferation (Figure 3)

### 3) TQ inhibits cell viability in a SCLC cell line

**Figure 5:**
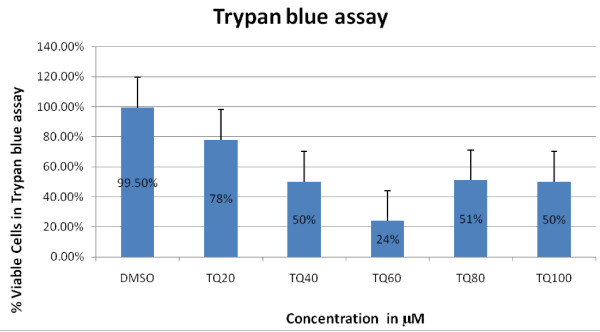
**Results of trypan blue cell viability assay using SCLC cell line NCI-H460 2 hrs after treatment with increasing concentration of TQ**. Cell viability decreased with increasing concentration of TQ and on average only 50% of cells were viable 2 hrs after treatment with various concentration of TQ.

Measurements of cell viability in a SCLC cell line NCI-H146 were determined using trypan blue assay. 24 hrs after exposure to TQ 20-100 uM on average only 50% of cells were viable as shown in (Figure 5).

### 4) Apoptosis assay

**Figure 6:**
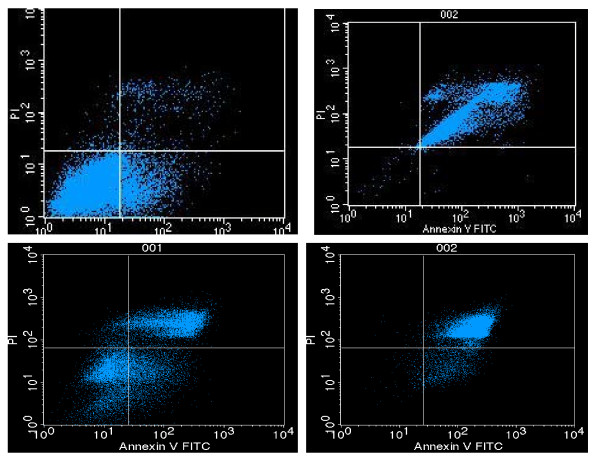
**Flowcytometry data showing apoptosis in both NSCLC (NCI-H460) and SCLC (NCI-H146) cell lines 24 hrs after treatment with TQ 100 μM. 87.59% of NCI-H460 cells and 88.1% of NCI-H146 cells were positive for Annexin-V 24 hrs after treatment with TQ**. Upper row represent NCI-H460 cells and Lower row NCI-H146. Left column represents control treated and the right column represents TQ treated.

To determine if the decrease in proliferation and viability were a result of TQ induced apoptosis in the two cell lines, cells were assayed by Annexin V staining after exposure to TQ. After 24 hours treatment with 100 μM TQ about 87.59% of NCI-H460 cells and 88.1% of NCI-H146 cells were positive for Annexin-V compared to 2.0% and 34.5% Annexin V positive cells in the DMSO treated cell (Figure 6)

### 3) TQ suppresses expression of cytokines involved in neo-angiogenesis

**Figure 7:**
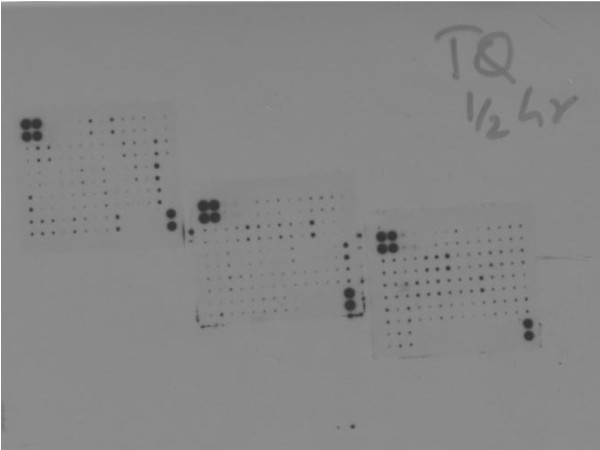
**Effect of TQ on release of various cytokines was determined using RayBio Human Cytokines Antibody Array C Series 2000**. TQ treated cell media was applied to cytokine membranes which were then exposed to a photographic film for 30 minutes and developed in a dark room. The three membranes represent various cytokines whose presence can be detected using this technique. Dots represent presence or absence of various cytokines which were then quantitated using image J Software expressed as mean integrated density.

**Figure 8:**
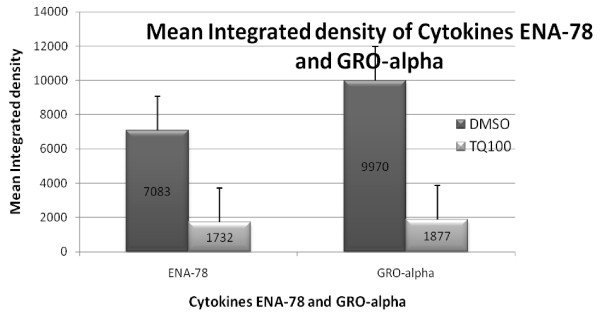
**TQ suppressed expression of cytokines ENA-78 and GRO-alpha significantly as compared to control**. These cytokines are implicated in neo-angiogenesis.

To assess the effect of TQ on release of various cytokines we assayed the culture media to determine if TQ affected expression of cytokines in NCI-H460 cell line. Of the panel of various cytokines measured using RayBio Human Cytokine Antibody Array C Series 2000, two cytokines ENA-78 and Gro-alpha were significantly lower in the media of cells exposed to 100 μM TQ as compared to control. The mean integrated density as measured by Image J Software for ENA-78 in the control treated group was 7083 as compared to 1732 in the TQ treated group and for Gro-alpha in the control group mean integrated density was 9970 as compared to 1877 in the TQ treated group (See figure 7-8)

### 4) TQ inhibits invasion in a Matrigel assay

**Figure 9:**
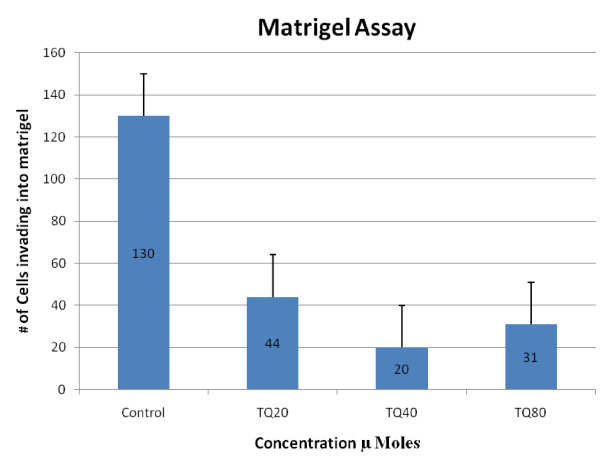
**Effect of TQ on invasion was assessed calculating number of cells invading into Matrigel**. TQ at increasing concentration inhibited cell invasion as compared to control.

Because of the known effects of TQ on decreasing specific cytokines production and the known effects of cytokines on tumor cell invasion, we determined the effects of TQ on tumor cell invasion as assayed by growth into Matrigel. TQ at three concentrations (20, 40 and 80 μM) significantly inhibited invasion as compared to control (P < 0.05). Inhibition of invasion was greatest at 40 μM where inhibition was 85% as compared to control (Figure 9)

### 5) Maximum tolerated dose (MTD) and toxicity study

Prior to determining the effect of TQ on the growth of xenografts we studied the toxicity of TQ and CDDP alone and in combination as noted in the Methods to determine the maximum tolerated dose (MTD). Besides vehicle control (group 1), TQ was administered (s.c) on Monday, Wednesday, and Friday for 3 weeks at doses of 5 mg/kg (group2), 10 mg/kg (group3) and 20 mg/kg (group4). CDDP was administered (i.p) once a week for 3 weeks at 5 mg/kg (group 5) alone or in combination with TQ at 5 mg/kg (group 6), 10 mg/kg (group 7) and 20 mg/kg (group 8). No mortality was observed in groups 1-6 though mice in group 6 lost 20-40% of body weight. 50% of the mice in group 7 and 75% in group 8 died. Histological analysis was performed on kidneys, liver, lung and heart of treated mice. There were no pathological abnormalities noted in the lungs and heart of any of the mice. In the analysis of the kidneys no pathological abnormality was observed in groups 1-4 (TQ treated alone) except for the presence of 5% focal proximal tubular damage noted in group 4 (TQ 20 mg/kg). In group 5 (CDDP5 mg/kg alone) there was proximal tubular damage noted in 20-30% of the samples. In the combination groups [7,8] diffuse tubular damage and acute tubular necrosis (ATN) was noted in 40-80% of samples. Mice in these groups also lost significant body weight and appeared dehydrated. This enhancement of nephrotoxicity may be related to poor by mouth intake and dehydration resulting in ATN. On the basis of these studies MTD was determined to be as follows: CDDP 2.5 mg/kg i.p. weekly along with TQ at 5 mg/kg and 20 mg/kg subcutaneously Monday, Wednesday and Friday for the xenograft study.

### 6) Mouse xenograft study

**Figure 10:**
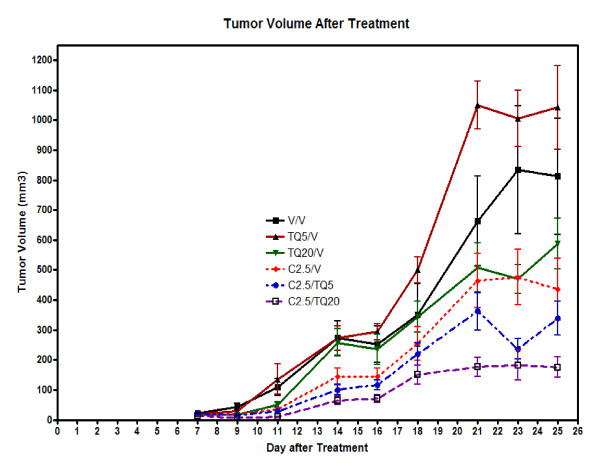
**Results of Mouse xenograft study**. Tumor volume with time: Change in tumor volume is shown in various treatment arms over the study period. Mice were treated with either s.c. TQ every Monday, Wednesday and Friday or CDDP i.p. once a week or combination.Mice in combination treatment arms (TQ20 mg/kg + CDDP 2.5 mg/kg) had the smallest tumor volume at the end of 3 week study period.

In the mouse xenograft study as described in methods section after 4 weeks of tumor growth no mortality occurred. However, the combination of TQ and CDDP had striking effects on tumor volume (Figure 10). TQ alone at 5 mg/kg was not active. The higher dose of TQ at 20 mg/kg demonstrated some activity and reduced tumor volume although the effect was marginally significant (p 0.075). Cisplatin alone at 2.5 mg/kg reduced tumor volume significantly (p < 0.001). The effect on tumor volume was greatest in the combination arms with significant reduction of tumor volume by 59% with the combination of (5 mg/kg TQ and 2.5 mg/kg CDDP) (p = 0.036) and by 79% with combination of (20 mg/kg TQ and 2.5 mg/kg of CDDP) (p = 0.0016).

**Figure 11:**
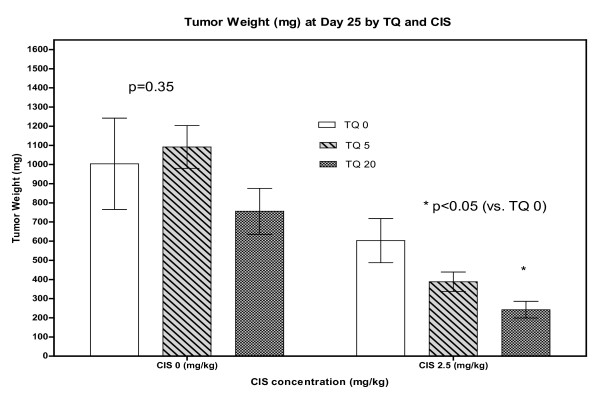
**Mean tumor weight at day 26 for each group**. (*) means significant inhibition by addition of TQ (p < 0.05).

The decrease in tumor volume was mimicked by a similar decrease in tumor weight in all treatment arms except TQ alone at 5 mg/kg (Figure 11)

### 7) TQ suppresses NF-κB expression in vivo

**Figure 12:**
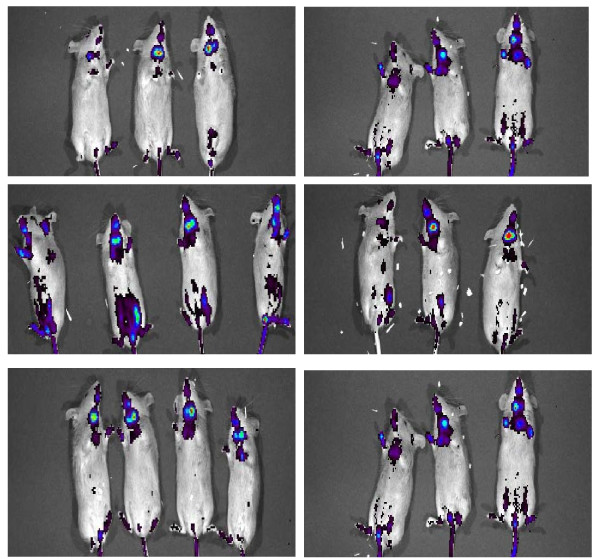
**LPS induced NF-κB expression using luciferase reporter mice**. Upper row: NF-κB expression pre-screen; Middle row NF-κB expression 3 hrs after LPS induction; Lower row NF-κB expression 24 hrs after LPS induction. Mice when pre-treated with TQ 5 mg/kg (Right column) showed less NF-κB expression at 3 hrs as compared to control treat mice (Left column). Level of NF-κB expression returned to baseline 24 hrs after exposure to LPS. The luminescence from luciferase was detected real time using an ultrasensitive camera IVIS 100 Imaging system. The luminescence intensity was quantitated in regions of interest (ROI) using Living Image^® ^3.0 software as shown in table 1.

TQ by itself had no effect on basal luciferase activity and NF-κB expression. (Figure 12 top panels). However, at 3 hrs after treatment with LPS the increased luminescence indicating activation of NF-κB was suppressed by prior treatment with TQ at 5 and 20 mg/kg as compared to control though this effect was not statistically significant (P < 0.10). This effect however was not observed at 24 hrs point interval, where most of luminescence had returned to baseline (Figure 12, Table 1)

**Table 1:** ROI values of Female Luciferase reporter mice*

	Control	TQ5 mg/kg	TQ20 mg/kg
Pre-Screen	15,490 +/- 2,108	17,155 +/- 8,957	11,990 +/- 3,031

LPS 3 hrs	176,375 +/- 63,901	89,457 +/- 24,084	75,923 +/- 33,793

LPS 24 hrs	23,978 +/- 5,501	24,177 +/- 6,830	39,823 +/- 13,631

NF-κB expression was measured by quantitating the luminescence intensity in regions of interest (ROI) using Living Image^® ^3.0 software (Caliper Life Sciences, Inc. Hopkinton, MA). (*) ROI values include +/- standard error (n = 3-4) obtained using Living Image Software version 3.0. ROI values are equal in the mice pre-treated with vehicle or TQ showing TQ has no effect on NF-κB expression. 3 hrs after LPS injection ROI values representing NF-κB expression are much lower in mice pre-treated with TQ at 5 and 20 mg/kg though not statistically significant (P < 0.10) as compared to control suggesting pre-treatment with TQ suppresses NF-κB expression. ROI return to baseline at 24 hrs in both groups.

### 8) Effect of TQ on expression of NF-κB in the xenografts

**Figure 13:**
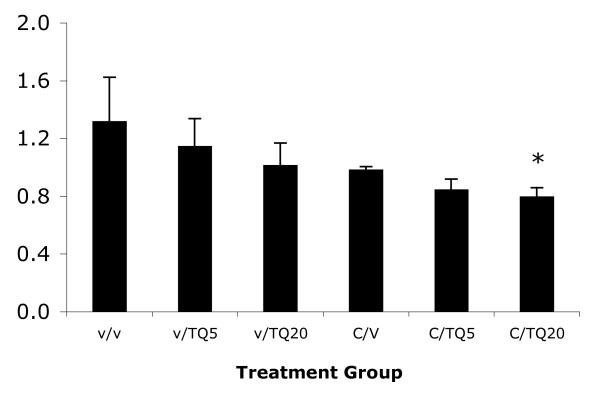
**Ratio of p-NF-kB/NF-kB in tumors**. The xenografts were evaluated for the effects of TQ on NF-κB expression with tumor lysates from xenografts analyzed by western blot for levels of phosphorylated NF-κB as a ratio of total NF-κB. V = Vehicle, TQ = Thymoquinone, C = CDDP at 2.5 mg/kg. Significant reduction in ratio of p NF-κB/NF-κB were seen in xenografts from mice treated with combination of TQ (20 mg/kg) and CDDP (2.5 mg/kg).

The xenografts were further evaluated for the effects of TQ on NF-κB expression with tumor lysates from xenografts analyzed by western blot for levels of phosphorylated NF-κB as a ratio of total NF-κB. Significant reduction in ratio of phosphor-Ser529 NF-κB/NF-κB were seen in xenografts from mice treated with combination of TQ (20 mg/kg) and CDDP (2.5 mg/kg) but not with TQ or CDDP alone (P < 0.05) (Figure 13)

## Discussion

We evaluated anti-neoplastic effect of TQ alone and more importantly in combination with CDDP in a NSCLC and a SCLC lung cancer cell line using both *in vitro *and *in vivo *techniques. TQ appeared to be active both in a NSCLC and a SCLC cell line. TQ inhibited proliferation of NSCLC cell line NCI-H460 and induced apoptosis. Similarly cell viability of SCLC cell lines NCI-H146 was decreased and cells underwent apoptosis after exposure to TQ. More importantly TQ acted synergistically with CDDP in a NSCLC cell line which is very encouraging. This inhibitory effect of TQ on lung cancer cell proliferation is not unique as recently TQ has been shown to inhibit growth of prostate, pancreatic and colon cancers [11] However, this is the first time that we have demonstrated anti-neoplastic effects of TQ in Lung Cancer using both a NSCLC and a SCLC cell line. Combination of TQ and CDDP is also unique and the results are encouraging as the two drugs have differing mechanism of action, the former being a cell cycle specific and the latter non-cell cycle specific. The dose of TQ used in these experiments may not be feasible in humans. Recently, Banerjee et al [21] have shown that more potent synthetic analogues of TQ can be prepared which can potentially be developed for future human use.

Besides anti-proliferative and pro-apoptotic effects TQ appears to affect tumor microenvironment. TQ reduced the release of two cytokines ENA-78 and Gro-alpha which are involved in inflammation and angiogenesis [22]. ENA-78 has been shown to be elevated in NSCLC surgical samples and correlates with tumor growth and vascularity [23]. ENA-78 and GRO belong to a family of ELR+ve CXC cytokines and are potent promoters of angiogenesis [24]. Similarly using Matrigel assay we were able to demonstrate that TQ inhibited invasion of NCI-H460 cells into Matrigel. Inhibition of tumor angiogenesis by TQ and its effects on invasion have recently been shown by others as well [25]. Thus TQ appears to be an agent that not only affects cell proliferation but may also influence the extra-cellular environment and immune system.

As far as toxicity from TQ is concerned there appears to be no significant toxicity demonstrated from use of TQ alone in our MTD study using female SCID mice. When TQ was used alone no mortality was observed, mice maintained their weight and no significant tissue damage was observed on histological analysis of liver and kidney. In the MTD study where a higher dose of CDDP (5 mg/kg) was used in combination with TQ mortality was observed in mice and most of the tissue damage was noticed to be in kidneys. It appears that the nephroprotective effects of TQ against CDDP as demonstrated in a previous study [12] were not reproduced in our model. The Combination of TQ with higher doses of CDDP also contributed to significant weight loss and apparent dehydration which may have resulted in worsening of kidney damage from CDDP and ultimately their demise. However, TQ alone or in combination with lower dose of CDDP (2.5 mg/kg i.p. weekly) did not appear to have any direct toxic effect on kidneys or liver. In the mouse xenograft model in combination with CDDP at 2.5 mg/kg there was weight loss but no mortality or tissue damage was observed on histological analysis of kidneys and liver.

In the mouse xenograft model TQ alone at 20 mg/kg was active. The combination of TQ and CDDP was more active than each agent alone. The combination of (20 mg/kg TQ and 2.5 mg/kg of CDDP) reduced tumor volume by 79% without additional toxicity to the mice. These results are very encouraging and consistent with our *in vitro *data and show that TQ and CDDP is an effective therapeutic combination in lung cancer.

TQ by itself was shown to suppress LPS-induced NF-κB activation in the NF-κB -Luc-Re mice which is consistent with known properties of TQ [16]. We substantiated this finding in the luciferase mouse with the analysis of p- NF-κB expression in lysates of the xenografts (Figure 13). The effect on NF-κB was present in the combination of CDDP and TQ as presumably the combination is blocking multiple pathways that activate the NF-κB. As altered NF-κB expression is implicated in CDDP resistance [14] the suppression of NF-κB by TQ may provide a mechanism for overcoming CDDP resistance which makes TQ an exciting compound to develop in combination with CDDP. Supporting our results is recent publication by Banerjee et al [26] in which TQ was shown to augment anti-tumor activity of Gemcitabine and Oxaliplatin in pancreatic cancer by down regulation of NF-κB.

Recently it has been shown that the effects of TQ are broad with the demonstration that TQ inhibits Polo like kinases (PLKs) [27], family of serine/threonine protein kinases which control critical steps in passage of cells through the M phase of the cell cycle [28].Also PLK1 is over expressed in NSCLC and has prognostic significance [29]. Therefore in using TQ in NSCLC we may target cell cycle not only at G1-S phase but also at M phase.

## Conclusions

Thus in conclusion, in this paper we have demonstrated anti-proliferative and pro-apoptotic activities of TQ in both a NSCLC and a SCLC cell lines. It also appears that there may be synergism between TQ and CDDP. This combination was active *in vivo *as demonstrated by the mouse xenograft sudy. By suppressing NF-κB, TQ may be able to overcome CDDP resistance and enhance its efficacy. Thus TQ or likely synthetic analogues of TQ should be developed for possible future human use not only in lung cancer but in possibly other tumor types as well.

## Abbreviations

(NSCLC): non-small cell lung cancer; (SCLC): small cell lung cancer; (TQ): Thymoquinone; (EAC): Ehrlic ascites sarcoma; (CDDP): Cisplatin; (PLKs): Polo like kinases; (ATCC): American type culture collection; (FBS): Fetal bovine serum; (DMSO): Dimethyl Sulfoxide; (MTT): 3-(4,5 Dimethylthiazol -2-yl)-2,5-dipheynyltetrazolium bromide; (SCID): Severe combined immunodeficiency; (NIH): National institute of health; (MTD): Maximum tolerated dose; (LPS): Lipopolysaccharide; (LPTA): light producing animal model; (TNF-α): Tumor necrosis factor-alpha; (CI): Combination index; (ANOVA): Factorial analyses of variance; (PI): Propidium iodide; (ATN): acute tubular necrosis; (NIH): National Institutes of Health; (NF-κB): Nuclear Factor kappa-B.

## Competing interests

The authors declare that they have no competing interests.

## Authors' contributions

SJ designed the study, carried out the experiments and wrote the manuscript. JG designed the study, provided guidance for the study and edited the manuscript. RS did the statistical analysis and made illustrations and graphs. SZ did histological analysis of tumor and tissue samples. MS helped with cell culture, western blot and mice studies. HK designed the study, carried out the experiments, wrote the manuscript and provided guidance at every step of the study. All authors have read and approved the final manuscript.

## Source of Funding

Syed H. Jafri received fellowship grant from Amgen Inc.
